# A Role for H_2_S in the Microcirculation of Newborns: The Major Metabolite of H_2_S (Thiosulphate) Is Increased in Preterm Infants

**DOI:** 10.1371/journal.pone.0105085

**Published:** 2014-08-14

**Authors:** Rebecca M. Dyson, Hannah K. Palliser, Joanna L. Latter, Grazyna Chwatko, Rafal Glowacki, Ian M. R. Wright

**Affiliations:** 1 Mothers and Babies Research Centre, Hunter Medical Research Institute, New Lambton Heights, NSW, Australia; 2 Discipline of Paediatrics and Child Health, School of Medicine and Public Health, University of Newcastle, Callaghan, NSW, Australia; 3 Illawarra Health and Medical Research Institute and Graduate School of Medicine, University of Wollongong, Wollongong, NSW, Australia; 4 School of Biomedical Sciences and Pharmacy, University of Newcastle, Callaghan, NSW, Australia; 5 Discipline of Public Health, School of Medicine and Public Health, University of Newcastle, Callaghan, NSW, Australia; 6 Department of Environmental Chemistry, Faculty of Chemistry, University of Lodz, Lodz, Poland; 7 Kaleidoscope Neonatal Intensive Care Unit, John Hunter Children’s Hospital, New Lambton Heights, NSW, Australia; Medical University Innsbruck, Austria

## Abstract

Excessive vasodilatation during the perinatal period is associated with cardiorespiratory instability in preterm neonates. Little evidence of the mechanisms controlling microvascular tone during circulatory transition exists. We hypothesised that hydrogen sulphide (H_2_S), an important regulator of microvascular reactivity and central cardiac function in adults and animal models, may contribute to the vasodilatation observed in preterm newborns. Term and preterm neonates (24–43 weeks gestational age) were studied. Peripheral microvascular blood flow was assessed by laser Doppler. Thiosulphate, a urinary metabolite of H_2_S, was determined by high performance liquid chromatography as a measure of 24 hr total body H_2_S turnover for the first 3 days of postnatal life. H_2_S turnover was greatest in very preterm infants and decreased with increasing gestational age (p = 0.0001). H_2_S turnover was stable across the first 72 hrs of life in older neonates. In very preterm neonates, H_2_S turnover increased significantly from day 1 to 3 (p = 0.0001); and males had higher H_2_S turnover than females (p = 0.04). A significant relationship between microvascular blood flow and H_2_S turnover was observed on day 2 of postnatal life (p = 0.0004). H_2_S may play a role in maintaining microvascular tone in the perinatal period. Neonates at the greatest risk of microvascular dysfunction characterised by inappropriate peripheral vasodilatation - very preterm male neonates - are also the neonates with highest levels of total body H_2_S turnover suggesting that overproduction of this gasotransmitter may contribute to microvascular dysfunction in preterms. Potentially, H_2_S is a target to selectively control microvascular tone in the circulation of newborns.

## Introduction

Hypotension and low cardiac output complicate the course of very preterm infants, mostly in the first 48 hrs [1]. Preterm male infants <29 weeks gestation have significantly lower mean arterial blood pressure at 12–24 hrs, require more inotropic support and have more resistant hypotension than females [2]. Measurement of superior vena caval flow suggests that abnormal regulation of vascular resistance plays a role [3], with inappropriate microvascular vasodilatation playing a major role in the development of hypotension. We previously demonstrated a significant relationship between microvascular dilatation, mean arterial pressure and poor outcome in a preterm neonatal population [4]. Furthermore, we identified a sexually dimorphic pattern in microvascular function - very preterm male infants have greater vasodilatation than female infants of the same gestational age at 24 h postnatal age [5], suggesting a sex-specific difference in the neonatal ability to control vascular tone. This may explain why males are more at risk of complications following premature birth – male preterm infants are at much greater risk of dying or suffering from chronic neurodevelopmental disability [6], [7]. The death rate for extremely preterm males is more than double that of females (26% vs. 12%) and male morbidity is reflected by a 13% increased length of stay and increased re-admissions within the first year of life [6], [8].

Recent evidence suggests that a mismatch between vasoconstrictor and vasodilator molecules in the preterm newborn may underlie these microvascular blood flow problems. For example, it has been shown that the relative expression of vasoconstrictors such as norepinephrine (highest in females and more mature infants), is associated with lower microvascular flow and greater physiological stability [9]. Conversely vasodilators, specifically markers for the gasotransmitters nitric oxide (NO) and carbon monoxide (CO), are highest in males and younger infants, i.e. those who exhibit increased vasodilatation [10]. However, the increases seen in NO occur outside the crucial early period of the first 24–48 hours. Furthermore, changes in CO only explain a proportion of the variance we measured in early vasodilator events. These results suggest another factor must play a significant role in aberrant vasodilation.

Hydrogen sulphide (H_2_S) has recently been of considerable interest in adult health and disease, with H_2_S identified as an important gaseous regulatory molecule with many biological and physiological roles, including synaptic modulation, neuroprotection and smooth muscle relaxation [11]. H_2_S is endogenously produced in amounts capable of causing vasodilatation, thus controlling blood pressure [12]. Despite compelling adult data, almost nothing is known about the role of H_2_S in the transitional circulation of the neonate. Its contribution to vasodilatation may be crucial for regulation and dysfunction of vascular tone in the neonate. A recent piglet study suggests that H_2_S may be important in at least the transitional cerebral circulation [13]. These data, combined with our observations on NO and CO, led us to hypothesize that H_2_S would contribute to the excessive vasodilatation observed in preterm neonates in the initial extrauterine period. Specifically, that H_2_S production would be greater in those infants at greatest risk of microvascular dysfunction – very preterm male neonates – and that levels would correlate with microvascular blood flow.

One of the major challenges in translating preclinical animal studies to humans is determining a robust, non-invasive method to measure disturbances in H_2_S signaling. Due to the short half-life and volatile nature of the gas, we pursued an indirect metabolic measure [14]. The metabolism of H_2_S can be divided into three distinct pathways: oxidation to sulphate, clearance by exhalation and reactions with metalloproteins and disulphide containing proteins. Oxidation to sulphate and subsequent excretion by the kidneys represents the major metabolic and excretory pathway, with urinary sulphate levels representing around 50% of an exogenous dose of H_2_S administered orally, subcutaneously, intraperitoneally or intravenously [15]. Sulphate is not a suitable analytical target as production from other sources of sulphur swamp the contribution of H_2_S [14]. Urinary thiosulphate, an intermediate of the breakdown of H_2_S to sulphate is routinely used as a marker of exposure to high H_2_S levels in cases of industrial or environmental exposure and thus represents a better analytical target when the issue is total body turnover of H_2_S [16], [17]. Such non-invasive measures, if sensitive enough to detect endogenous H_2_S production, are suitable for clinical monitoring where tissues for analysis of enzyme expression and activity are not available.

The aim of the present study was to measure H_2_S output (as thiosulphate) in newborns, characterise levels in relation to gestational age, postnatal age and sex and to assess whether H_2_S turnover was associated with microvascular blood flow; the latter having previously been shown to correlate strongly with clinical illness severity and physiological stability in the sick newborn human infant [4].

## Materials and Methods

### Subjects

The “Cardiovascular Adaptation of the Newborn Study 2 (CANS2)” was conducted at the John Hunter Children’s Hospital, Newcastle, Australia between September 2008 and April 2011. This study was approved by the Human Research Ethics Committees of the Hunter New England Area Health Service and the University of Newcastle. Parental informed, written consent was obtained prior to investigation. Recruitment was stratified a priori to neonates born at 28 weeks gestational age (GA) or less (very preterm neonates), neonates born at 29–36 weeks (preterm neonates) and those born at 37+ weeks completed gestation (term neonates). Hypoxic ischemic encephalopathy, congenital malformations, chromosomal disorders or known congenital infection excluded admission to this study. Methods for recording of clinical and physiologic variables have been reported previously [4].

### Microvascular Studies

Laser Doppler is the best-established method of assessing peripheral microvascular function [18]. Low-intensity laser light is reflected by moving cells in the peripheral cutaneous circulation, enabling measurement of both number and velocity of blood cells moving through the skin microcirculatory bed, giving a measure of peripheral microvascular blood flow in Perfusion Units (PU). For laser Doppler assessment we used a Periflux 5001 laser Doppler (Perimed AB, Jarfalla, Sweden) with a temperature-regulated probe (Probe 457, Perimed) sited on the lateral aspect of the neonates’ lower limb. Investigations were performed at 6, 24 and 72 hr postnatal age as previously described [5]. Briefly, basal peripheral microvascular blood flow was recorded for 5 minutes before lower limb blood flow was occluded using a sphygmomanometer cuff to produce a 1-minute period of absent flow. This allowed biological zero to be obtained, which was subtracted from the basal blood flow in each experiment, allowing comparison between different studies and subjects.

A significant interaction of gestational age and sex was observed for total body turnover of H_2_S. In very preterm (24–28 wk) neonates, H_2_S turnover in the first three days of postnatal life was higher in males than in females (p = 0.04; Figure 3). Post hoc analysis revealed this was due to higher H_2_S turnover in very preterm males compared to females of the same gestational age group on both day 1 (p = 0.01) and day 2 (p = 0.04) of postnatal life.

### Urine collection and Analysis

Twenty-four hour urine samples were collected on days 1–3 of postnatal life as previously described [9]. Disposable diapers of the appropriate size containing a pure cellulose pad were used for urine collection. Diapers were changed every 4–6 hours as clinically appropriate and collected in a plastic bag at 4°C until completion of a 24 hr collection period then folded inside out and the urine extracted using a specially constructed press. Each 24 hrs of pooled specimens were stored at −80°C and spun before analysis. Exact 24 hr urinary output was calculated by weighing diapers before and after use. As humidity can contribute to diaper weight, the degree and length of time in humidity were recorded and adjustments were made as appropriate [19]. The corrected values were used for 24 hr output values for analysis. Assessment of urinary creatinine was carried out by Hunter Area Pathology Services using the CREA method (a modification of the kinetic Jaffe reaction) with Flex reagent cartridges (Siemens Healthcare Diagnostics Inc., Camberley, United Kingdom) on the Dimension Vista System (Siemens).

### Thiosulphate measurement

Determination of thiosulphate in neonatal urine was based on derivatisation with 2-chloro-1-methylquinolinium tetrafluoroborate and separation and quantification of derivative by reversed-phase liquid chromatography. A Hewlett-Packard 1100 Series system (Waldbronn, Germany) with a Zorbax SB-C18 (150 mm×4.6 mm, 5 µm) column (Agilent Technologies), controlled by ChemStation software (Hewlett-Packard) was used as described previously [20]. Briefly, isocratic elution, with a mobile phase consisting of a mixture of acetonitrile and water in the ratio of 60∶40 (v/v), was used. Temperature was set at 25°C, the flow-rate 1 mL/min and the detector wavelength 375 nm. Identification of peaks was based on comparison of retention times and diode-array spectra, taken at time of analysis, with corresponding sets of data obtained for authentic compounds.

Urinary creatinine is a commonly used index to adjust for renal function, however, the creatinine ratio often used in adults may be unsuitable for neonates because of low excretion of creatinine in infants [21]. Early studies observed a six-fold variance in creatinine excretion between individuals less than 1 year postnatal age. Furthermore, a wide variation in daily creatinine output was also observed in infants, and this has been attributed to individual metabolic variation [22]. In order to overcome this, 24 hr excretion values for urinary thiosulphate were calculated relative to 24 hr urinary output (mL/24 hr) and body weight (kg) in this study. Therefore, total body turnover of H_2_S is expressed as nmol/24 hr/kg for day 1, 2 and 3 of postnatal life. The same relationships, as presented for the output/kg analyses, were observed when corrected to 24 hr urinary creatinine and are thus not reported.

Day 2 H_2_S turnover was significantly correlated with microvascular blood flow at 24 h postnatal age (p = 0.0004, *r* = 0.37). This was largely due to preterm male neonates (p = 0.04, *r* = 0.43; figure 4) and was not observed in female neonates of the same gestational age group (p = 0.97, *r* = −0.01) or term neonates (p = 0.82, *r* = −0.08). No correlation was observed in very preterm neonates alone (p = 0.28, *r* = 0.19), despite neonates in this group having the highest levels of both microvascular blood flow and H_2_S turnover. In the preterm neonatal group (29–36 weeks GA), H_2_S turnover was negatively correlated with both systolic (p = 0.01, *r* = −0.39) and diastolic (0.04, *r* = −0.33) blood pressure on day 2 of postnatal life. No relationship was observed in the very preterm neonatal group (systolic: p = 0.33, *r* = 0.17; diastolic: p = 0.25, *r* = 0.20).

### Statistical methods

Stata 11 for MacOSX (StataCorp LP, Texas, USA) was used for statistical analyses. Stata 11 and Prism 5 for MacOSX (GraphPad Software Inc., La Jolla, CA) were used for generation of graphs. Data presented as median (range) unless otherwise stated. Differences between gestational age groups were analyzed by Kruskal-Wallis multiple comparisons test. Sex differences were analyzed by Mann-Whitney U-test. Thiosulphate levels between days for individuals were analyzed using Friedman repeated measures ANOVA for non-parametric data and random effects generalized least squares regression with bootstrapping. For correlations, data was analyzed using Spearman *r* correlation or transformed using natural logarithm (log_e_) and analyzed using Pearson correlation, depending on the normality of data distribution.

## Results

A total of 136 infants were recruited to the CANS2 Study. Due to study design, only neonates with urine available for all three days were included in this study. Therefore, 90 neonates were studied for H_2_S turnover during circulatory transition. Their clinical characteristics are shown in Table 1. Early discharge policy led to fewer term infants but most clinical differences were due to the effects of a priori allocation to different gestational age groups or the known sexual dimorphism effects of fetal growth [23].

**Table 1:** Clinical Characteristics of Neonates.

	Very Preterm Group		Preterm Group		Term Group	
	Female (n = 20)	Male (n = 16)	Female (n = 19)	Male (n = 24)	Female (n = 6)	Male (n = 5)
Gestation (wk)	26 (24–28)	26.5 (24–28)	32 (29–35)	31 (29–35)	38.5 (38–41)	39 (38–43)
Birth weight (kg)	0.87 (0.6–1.4)	1.0 (0.6–1.4)*	1.76 (1.0–2.4)	1.65 (0.9–2.8)	3.34 (3.0–4.0)	4.2 (3.3–4.3)
Multiple Birth (n, %)	2 (10%)	7 (44%)*	6 (32%)	11 (46%)	0	0
Completed antenatal steroids (n, %)	14 (70%)	13 (81%)	12 (63%)	18 (75%)	1 (16%)	0
Maternal Chorioamnionitis (n, %)	2 (10%)	1 (6%)	1 (5%)	2 (8%)	0	0
Maternal Smoking (n, %)	4 (20%)	3 (19%)	4 (21%)	5 (21%)	0	0
Pregnancy-induced Hypertension (n, %)	0	2 (13%)	2 (11%)	2 (8%)	0	0
Small for gestational age (n, %)	0	0	0	2 (8%)	1 (16%)	0
Vaginal delivery (n, %)	10 (50%)	7 (44%)	11 (58%)	11 (46%)	4 (67%)	4 (80%)
5-min APGAR score	8 (4–10)	8 (5–10)	9 (5–10)	9 (5–10)	9	9 (9–10)
CRIB II score	11 (8–15)	10 (7–16)	3 (1–8)	4 (1–8)	3	-
Mean Blood Pressure at 24 h (mmHg)	35 (24–43)†	36 (31–46)	50 (30–81)	39.5 (30–81)*	-	-
Mechanical ventilation (hr)	0 (0–20)	0 (0–24)	0 (0–10)	0 (0–2)	-	-
CPAP (hr)	3 (0–14)	1.5 (0–20)	0 (0–19)	0 (0–16)	-	-
Patent Ductus Arteriosus (n, %)	10 (50%)	6 (38%)	1 (5%)	3 (13%)	0	0
Sepsis (n, %)	6 (30%)	8 (50%)	3 (16%)	2 (8%)	0	0
IVH > grade II (n, %)	1 (5%)	2 (13%)	1 (5%)	0	0	0
Death (n, %)	4 (20%)	2 (13%)	0	0	0	0

Data presented as median (minimum-maximum) or number (%). APGAR Score – scores 7 and above are generally regarded as normal, 4 to 6 fairly low and 3 and below critically low; CRIB II Score – Clinical Risk Index for Babies II, higher scores reflect poorer physiological stability; CPAP – Continuous Positive Air Pressure respiratory support; Patent Ductus Arteriosus refers to a hemodynamically significant duct diagnosed in first 72 hrs; IVH – intraventricular hemorrhage greater than grade II (significant IVH); Mean Blood Pressure reported is that at 24 h postnatal age and was not assessed in term controls; Death is those infants that survived to 72 h postnatal age but died prior to discharge.

*significantly different from females of the same gestational age group p<0.05;

† significantly different from preterm neonates, within sex.

### Peripheral microvascular blood

Baseline microvascular blood flow demonstrated a significant inverse relationship with gestational age at 6 hr (p<0.0001, *r* = −0.54), 24 hr (p<0.0001, *r* = −0.63) and 72 hr (p = 0.0003, *r* = −0.38) postnatal age. As in previous studies [4], there was a strong positive correlation between microvascular blood flow and Clinical Risk Index for Babies (CRIB) II Score at 24 h postnatal age (p = 0.0008, *r* = 0.41). When split for sex, this relationship was significant only in males (p = 0.0003, *r* = 0.60). Also in line with previous studies, baseline peripheral microvascular blood flow exhibited significant relationships with measures of cardiovascular function at 24 hr postnatal age. In neonates ≤36 weeks gestational age at birth, baseline microvascular blood flow was inversely related to mean arterial blood pressure (p = 0.0034, *r* = −0.34). This relationship with microvascular blood flow was observed for both systolic (p = 0.0013, *r* = −0.37) and diastolic pressure (p = 0.0007, *r* = −0.39). When analyzed by sex, this relationship was observed in females only for all three measures (mean arterial pressure: p = 0.0021, *r* = −0.50; systolic blood pressure: p = 0.0016, *r = −*0.51; and diastolic pressure p = 0.0049, *r* = −0.46).

### H_2_S total body turnover

**Figure 1:**
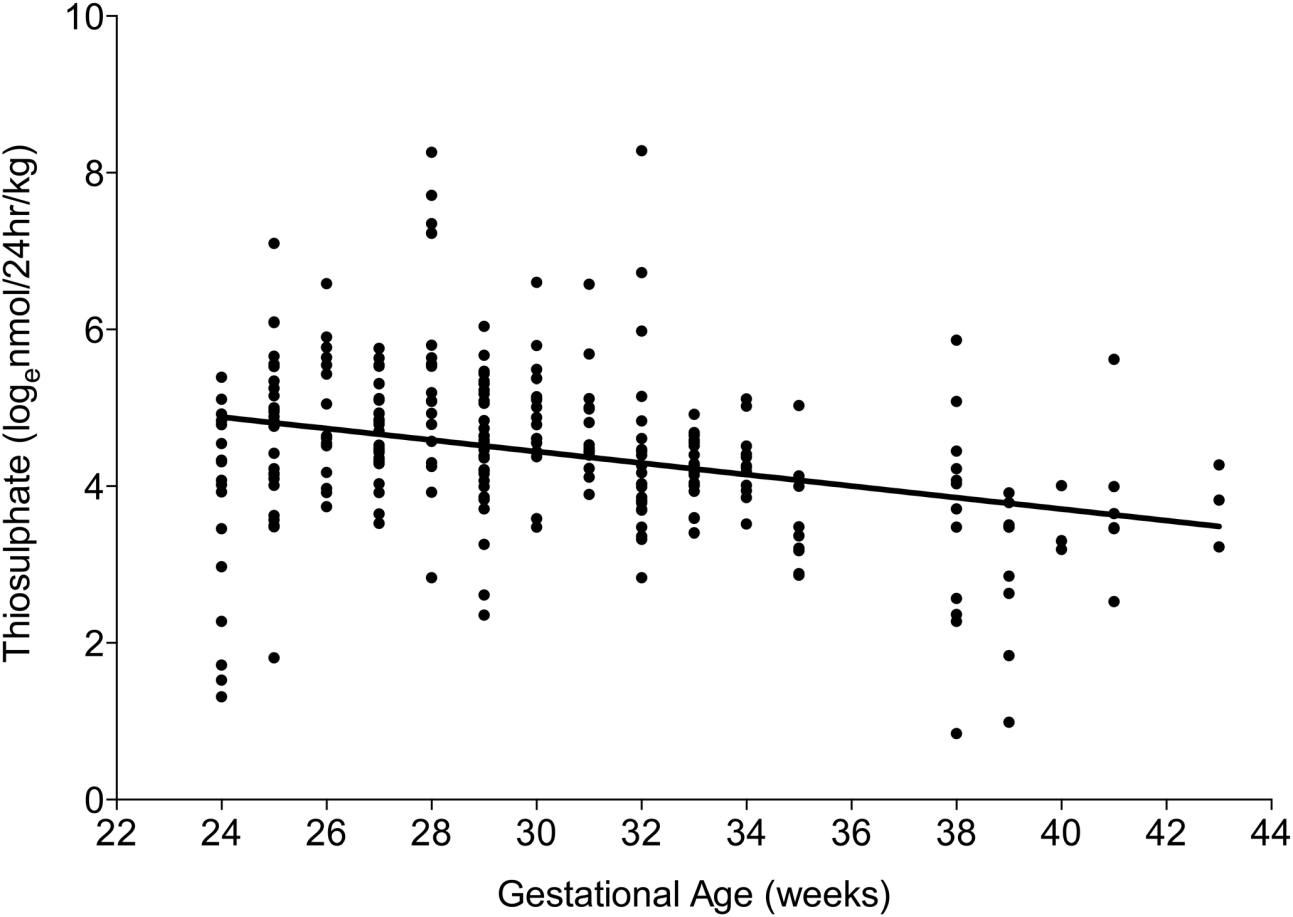
H_2_S turnover over the first 72 hours of postnatal life. Thiosulphate, as a marker of H_2_S turnover, as measured in urine samples over the first 3 days of life was lowest in term neonates and increased with increasing prematurity (Pearson correlation; p<0.0001, *r = −*0.32).

**Figure 2:**
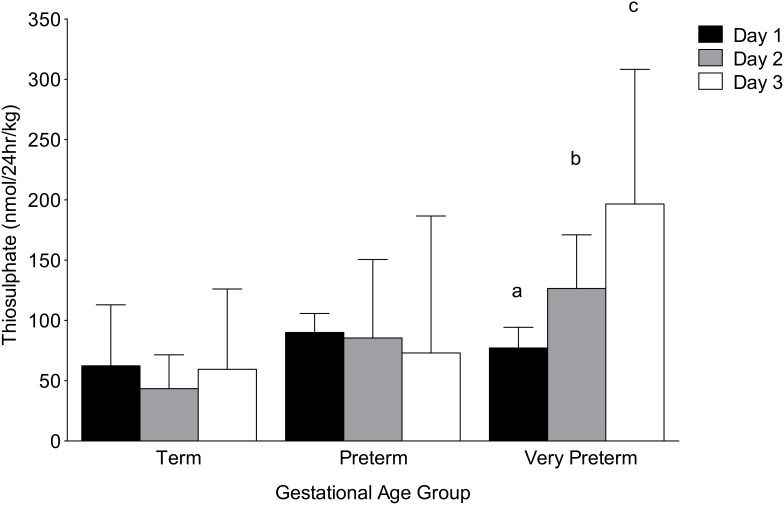
Urinary thiosulphate levels over the first three days of life. H_2_S turnover was stable across the first three days of life in term and preterm neonates. In very preterm neonates, levels rose significantly over the first 72 hours of life (median±IQR). ^a-b-c^p<0.0001 significant difference across days in very preterm gestational age group (Friedman repeated measures ANOVA for non-parametric data).

H_2_S turnover, as measured by urinary thiosulphate, over the first 72 hr of postnatal life was highest in the very preterm neonates, decreasing with increasing gestational age at birth (p = 0.0001; Figure 1). H_2_S turnover on day 1 was lower in term neonates (44.4 nmol/24 hr/kg; p = 0.03) but was comparable between preterm and very preterm neonates (preterm: 80.6 nmol/24 hr/kg, very preterm: 66.4 nmol/24 hr/kg; p = 0.17). H_2_S turnover remained stable across the first 72 hours of life in term and preterm neonates. However, in very preterm neonates, total body turnover of H_2_S increased significantly from day 1 to 3 (p = 0.0001; Figure 2).

**Figure 3:**
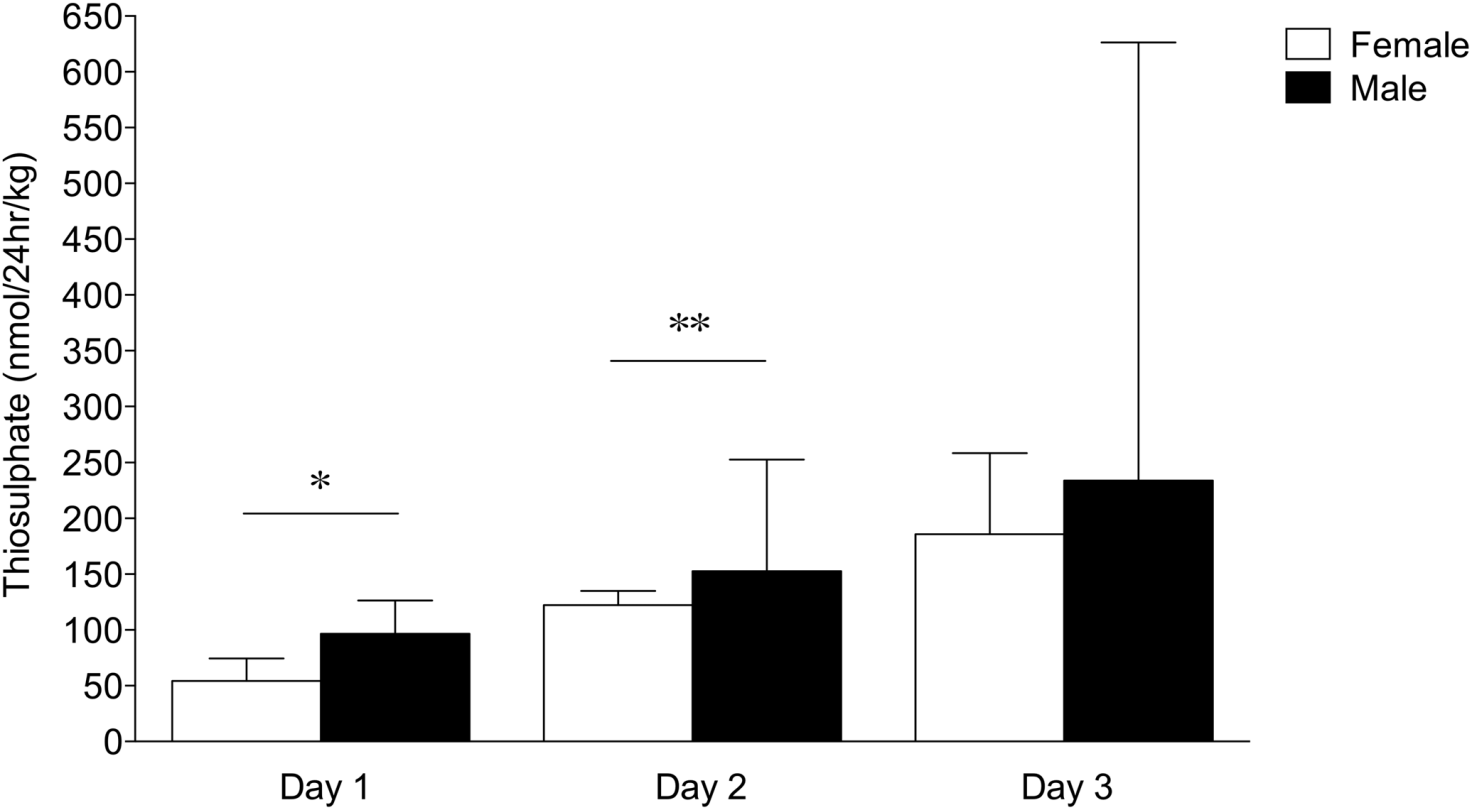
Sex differences in thiosulphate levels in very preterm neonates in early postnatal life (median±IQR). H_2_S turnover, measured as urinary thiosulphate excreted per day per kg body weight, was significantly higher in males than females on both day 1 (*p = 0.01) and day 2 (**p = 0.04) of postnatal life (Friedman repeated measures ANOVA for non-parametric data).

**Figure 4:**
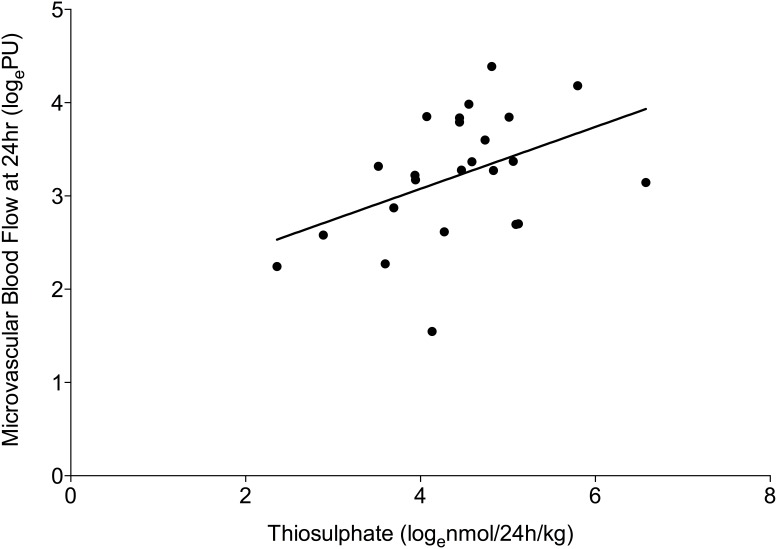
Relationship between baseline microvascular blood flow at 24 hr and H_2_S turnover on day 2 of postnatal life. H_2_S turnover (measured as urinary thiosulphate) was significantly correlated with baseline microvascular blood flow in preterm male neonates 29–36 wk GA (Pearson correlation; p = 0.04, *r* = 0.43). No relationship was observed for females of the same gestational age, very preterm neonates (24–28 wk GA) or term neonates (37+wk GA).

No significant effect of the other clinical variables outlined in Table 1 was seen on microvascular blood flow or H_2_S turnover after adjusting for the main effects of gestational age and sex.

## Discussion

We have shown, for the first time, that in human infants there is evidence of a role for the gasotransmitter H_2_S in the control of the microvasculature. We have presented data showing being an at-risk preterm infant increases the output of the major product of H_2_S metabolism (thiosulphate). We have shown this to be related independently to all the major risk factors for poor outcome independently: gestational age, postnatal age and male sex.

Thiosulphate levels are comparable between preterm and very preterm neonates for the first 24 h of postnatal life. However whilst they remain low in older preterm neonates, levels increase significantly from day 1 to day 2, and again from day 2 to day 3 in very preterm neonates. This suggests that very preterm neonates are not born with inherently higher levels of H_2_S production, but that H_2_S production increases significantly following birth. Potential triggers for this would include oxidative stress [24] or inflammation, both of which have been implicated in changes following preterm delivery [25]. The findings that H_2_S turnover increased postnatally in the very preterm group, but not in the preterm group, and that microvascular blood flow was significantly greater in the former also suggests that there is a physiological difference between neonates born very preterm and those born at later gestational ages. It also suggests that there are significant developmental changes in the regulation of the gasotransmitter production pathway throughout gestation and early postnatal life.

The positive relationship of H_2_S turnover with microvascular blood flow and the inverse relationship with blood pressure in more mature neonates suggests a physiological role of H_2_S in this age group, perhaps as a counter to the overarching constrictive balance [9], or as a reflection of an organ specific vascular dilatation, such as in the pulmonary circulation [26], [27]. This remains an area of speculation and more research is required.

We observed a significant relationship between microvascular blood flow at 24 hr postnatal age and day 2 urinary thiosulphate levels in males <37 weeks gestational age. This relationship was not present in the very preterm group alone, despite these neonates having the highest microvascular blood flow and the highest thiosulphate excretion at this time. The lack of a conclusive relationship suggests that dysregulation of microvascular tone may not solely be the result of disturbances in H_2_S production, but may result from an imbalance of vasoconstrictors and vasodilators, including H_2_S. Previous work has shown that the other gasotransmitters, NO and CO are both produced in, and exert some effect on, the transitional microcirculation of preterm neonates [10]. Further, it is possible that as in the state of neonatal and adult shock a tight relationship between blood pressure, microvascular tone and mediators is lost below a threshold level [28], [29].

It is becoming increasingly evident that microvascular function is not controlled by the activity of these gasotransmitters working in isolation, but by the interaction of all three, underlining the complexity of hemodynamic microvascular control. It appears that CO and H_2_S both play an important role during circulatory transition and following the immediate extrauterine period, while NO is critical for maintaining basal microvascular tone later, with significant effects of nitric oxide on hemodynamic status observed in neonates at 7 days postnatal age [10], [30]. In addition to the vasodilators, a number of vasoconstrictive mediators also play a role in the regulation of microvascular tone in the newborn. Microvascular dysregulation in the preterm newborn is associated with both impaired vasoconstriction [9] and abnormal peripheral dilatation [10], contributing to cardiovascular compromise and poor outcome, highlighting the importance of balance in homeostasis and the profound effect imbalance can have on physiological stability. Understanding the control of blood flow in the perinatal period is a critical step for the development of therapeutic strategies for the management of the newborn at risk of cardiovascular compromise.

A number of groups suggest that H_2_S may play a role in central cardiac function. In adults and experimental myocardial ischemia-reperfusion models, H_2_S protects against cellular injury [31], cardiomyocyte loss [32] and arrhythmias [33], reduces infarct size [34], and improves microvascular reactivity [35] and cardiac contractility [36]. Importantly, dysregulation of the H_2_S pathway in adults has been implicated in a number of disease states, including coronary heart disease and hypertension where decreased plasma H_2_S levels, theoretically leading to a relative state of vasoconstriction, correlate with disease severity [37], [38].

H_2_S production is induced by shock states (inflammatory [39], [40], circulatory [41], septic [42], hemorrhagic [43] and endotoxic [44], [45]) and results in marked inflammation and injury. These studies highlight the potent pro-inflammatory properties of H_2_S and provide evidence for a pivotal role of H_2_S in the pathophysiology of conditions associated with both local and systemic inflammation and circulatory dysfunction. However, we did not see any differences in H_2_S turnover between neonates with or without sepsis, despite elevated levels expected in septic patients. This may be due to the small numbers in the septic group (n = 19 across sexes and gestational age groups) or the fact that many neonates in the “non-septic” group may have subclinical levels of sepsis and may have slightly elevated H_2_S production, confounding the comparison. Further investigation and comparison between a larger population of confirmed healthy and septic newborns is required.

H_2_S is produced from the amino acids cysteine, homocysteine and cystathionine by the activity of cystathionine-γ-lyase (cystathionase; CSE, EC 4.4.1.1), cystathionine-β-synthase (CBS, EC 4.2.1.22) or 3-mercaptopyruvate sulphurtransferase (MPST, EC 2.8.1.2).[46] Considerable research has been conducted into the activity of CSE, the enzyme responsible for converting cystathionine to cysteine via the transsulphuration pathway in the preterm neonate. CSE activity is gestational- and postnatal-age dependent, with significantly higher levels of hepatic activity in full term than preterm newborns [47]. This hepatic activity is known to increase during fetal-to-neonatal transition, such that the newborn exhibits significantly higher activity compared to the fetus, with significantly increased levels of both mRNA and protein [48], [49]. The results of the present study, which show a high total body turnover of H_2_S in the initial extrauterine period, are at odds with earlier reports of CSE activity being lower in preterm than term newborns [47]. This may be a result of tissue specific regulation: previous studies have looked only at hepatic activity, whereas our results reflect total body H_2_S turnover. These earlier studies looked at the conversion of cystathionine to cysteine as the end point of the CSE mediated pathway, however, CSE is also responsible for the further downstream metabolism of cysteine which results in H_2_S production, and this second role, which was not previously studied, may result in an accelerated breakdown of cysteine in the preterm neonate, contributing to high levels of H_2_S during circulatory transition. Additionally, high H_2_S production could also occur in the absence of high endogenous cysteine as CSE can also use homocysteine and cystathionine as substrates to produce H_2_S [50].

It is also possible that the CSE arm of the H_2_S production pathway is not the predominant player in H_2_S production during the perinatal period. CBS and MPST are also known to catalyze the production of H_2_S. The concept of H_2_S production enzymes following a tissue-specific expression profile is currently being challenged. Until recently, it was believed that the major source of H_2_S in the vasculature was CSE. More recently it has been shown that CBS, the enzyme originally thought to be responsible for H_2_S production predominantly in the brain and nervous tissue, is also expressed in the vasculature, and a third, more recently discovered pathway for synthesizing H_2_S via MPST has also been identified in rodent vasculature [51]. Little is known about these three production pathways in the human vasculature or in the neonatal period. Neonatal CBS deficiency manifests as homocystinuria associated with neurodevelopmental delay and skeletal and vascular abnormalities, highlighting the importance of this pathway not only in the brain, but in a number of other systems, including the vasculature [52]. Clearly future studies will need to address all enzymes in the H_2_S production pathway. Further elucidation of the activity of these enzymes will help to define possible intervention strategies.

A limitation of our study is that whilst we have demonstrated a clear correlation between outcome, microvascular blood flow and H_2_S production, this does not prove causation. Nevertheless we believe that our study provides strong clinical data that this pathway is involved in microvascular tone regulation during circulatory transition. Furthermore, it highlights both the need for mechanistic studies utilising available animal models [13], [53], and alternative measures of H_2_S production. Exhaled H_2_S may provide us with better temporal resolution of H_2_S production [54].

These results provide the first evidence that H_2_S may play a role in maintaining microvascular tone of the neonate in the perinatal period. Thiosulphate levels (as a marker of total body turnover of H_2_S) were found to be highest in those neonates at greatest risk of microvascular dysfunction characterized by inappropriate peripheral vasodilatation – very preterm male neonates born at 28 weeks completed gestation or less, suggesting that overproduction of H_2_S may contribute to microvascular dysfunction in neonates and thus to both their mortality and long term morbidity. The hydrogen sulphide pathway potentially represents a novel therapeutic target for the selective control of vascular tone and development during fetal-to-neonatal circulatory transition, which may help to reduce cardiovascular compromise following preterm birth, leading to better short- and long- term outcomes for this vulnerable group.
